# Polysaccharides from Traditional Chinese Medicines: Extraction, Purification, Modification, and Biological Activity

**DOI:** 10.3390/molecules21121705

**Published:** 2016-12-13

**Authors:** Yun Chen, Fangke Yao, Ke Ming, Deyun Wang, Yuanliang Hu, Jiaguo Liu

**Affiliations:** Institute of Traditional Chinese Veterinary Medicine, College of Veterinary Medicine, Nanjing Agricultural University, Nanjing 210095, China; 2014207027@njau.edu.cn (Y.C.); 2015107098@njau.edu.cn (F.Y.); 2014107093@njau.edu.cn (K.M.); dywang@njau.edu.cn (D.W.); ylhu@njau.edu.cn (Y.H.)

**Keywords:** Traditional Chinese Medicine, polysaccharide, extraction, modification, biological activity

## Abstract

Traditional Chinese Medicine (TCM) has been used to treat diseases in China for thousands of years. TCM compositions are complex, using as their various sources plants, animals, fungi, and minerals. Polysaccharides are one of the active and important ingredients of TCMs. Polysaccharides from TCMs exhibit a wide range of biological activities in terms of immunity- modifying, antiviral, anti-inflammatory, anti-oxidative, and anti-tumor properties. With their widespread biological activities, polysaccharides consistently attract scientist's interests, and the studies often concentrate on the extraction, purification, and biological activity of TCM polysaccharides. Currently, numerous studies have shown that the modification of polysaccharides can heighten or change the biological activities, which is a new angle of polysaccharide research. This review highlights the current knowledge of TCM polysaccharides, including their extraction, purification, modification, and biological activity, which will hopefully provide profound insights facilitating further research and development.

## 1. Introduction

**Figure 1:**
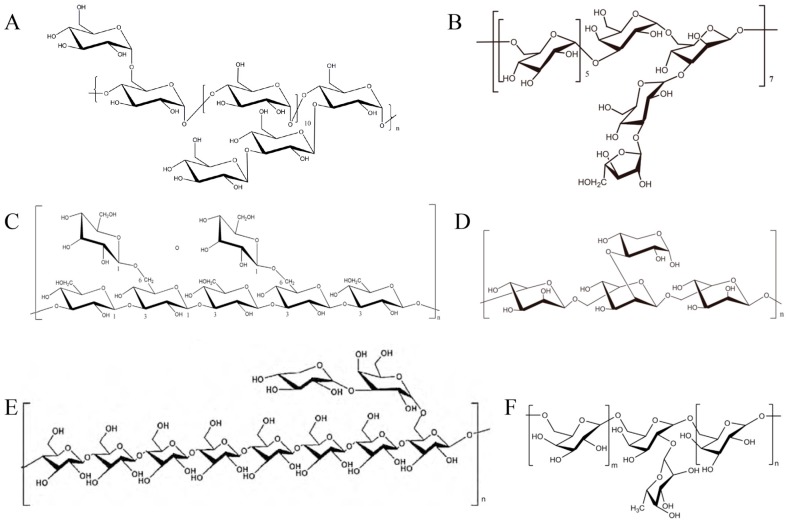
Examples of structures of some TCM polysaccharides. (**A**) *Bush sophora* root polysaccharide, whose average molecular weight is 2.24 × 10^4^ Da [4]; (**B**) *Euphorbia fischeriana* polysaccharide, whose average molecular weight is 1.12 × 10^4^ Da [5]; (**C**) *Lentinula edodes* polysaccharide [3]; (**D**) *Lactarius deliciosus* Gray polysaccharide, whose average molecular weight is 1.1 × 10^4^ Da [6]; (**E**) *Tricholoma matsutake* polysaccharide, whose average molecular weight is 8.89 × 10^4^ Da [7]; and (**F**) *Hericium erinaceus* polysaccharide, m + n = 3, whose average molecular weight is 1.5 × 10^4^ Da [8].

For thousands of years, Traditional Chinese Medicine (TCM) has been the most important therapeutic method in China and even the whole of East Asia. As we know, the compositions of traditional Chinese medicines are complex, and their active ingredients are often polysaccharides, saponins, flavonoids, polyphenols, and polypeptides [1]. Many extracts from TCM, such as artemisinin, arsenic trioxide, and curcumin display important roles in medicine and have been used in the clinic [2]. Polysaccharides from TCM have been a research hotspot recently. They are a kind of biomacromolecule composed of ten or more monosaccharides (Figure 1) whose structure and sugar composition vary. It is reported that at least thirty polysaccharides have been studied in standard clinical trials [3].

**Figure 2:**
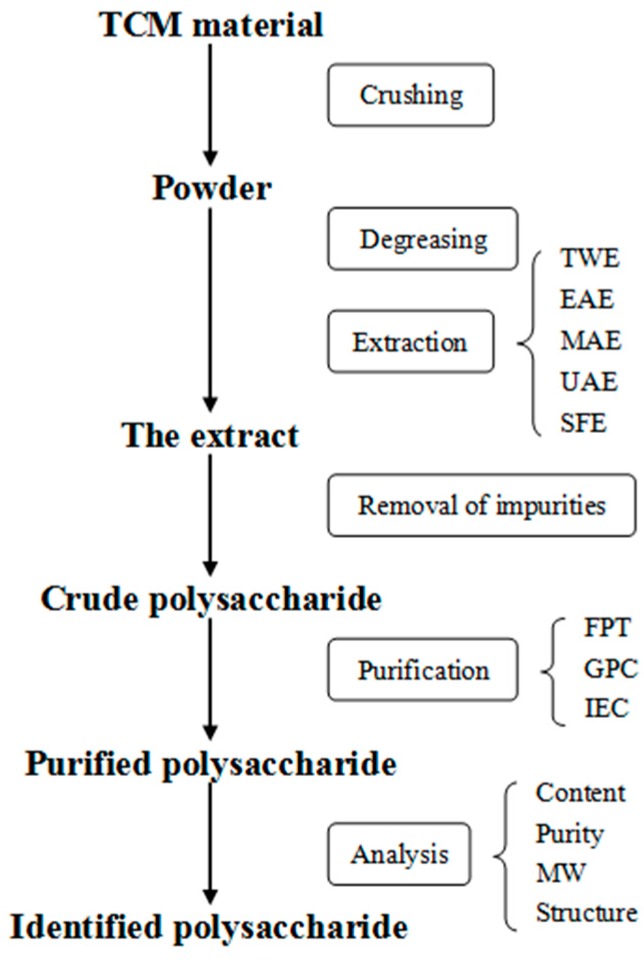
The flowchart of the extraction, purification, and analysis of polysaccharides. TWE: traditional water extraction, EAE: enzyme-assisted extraction, MAE: microwave-assisted extraction, UAE: ultrasonic-assisted extraction, SFE: supercritical fluid extraction, FPT: fractionated precipitation technique, GPC: gel permeation chromatography, IEC: ion exchange chromatography, MW: molecular weight.

Studies on TCM polysaccharides generally focus on their extraction, purification, structure, modification, and biological activity. Extraction methods of TCM polysaccharides are various and always influence the variety and characteristics of the final extracted products [3]. The most widely used method is hot water extraction [3,9], although some polysaccharides are not soluble in hot water and therefore not extracted by this technique. Consequently, many new methods and techniques have been applied to develop efficient extractions (Figure 2). No matter the extraction method, the purity of the polysaccharide is generally low. Consequently, purification and structural determination studies are important (Figure 2). The biological activities of polysaccharides from TCMs are varied and, in general, one polysaccharide always expresses several biological activities. For example, *Astragalus* polysaccharide exhibits immunoregulatory [10], anti-oxidative [11], antiviral [12], and anti-tumor [13] activities. Currently, many studies have focused on the immunity-modifying, antiviral, anti-inflammatory, anti-oxidative, and anti-tumor activities of TCM polysaccharides [14,15,16]. Moreover, chemical modifications can heighten or change the biological activities of TCM polysaccharides [17,18,19] and have attracted more and more attention. As we know, the extraction and purification of polysaccharides are so complicated that it is quite difficult to obtain pure active polysaccharides. From another perspective, this suggests that the extraction and purification also have significant influence on the modification and biological activity of polysaccharides. Different extraction methods and purified fractions may lead to different biological activities of polysaccharides, thereby influencing the modification. Thus, extraction and purification are the necessary prerequisites for the analysis of biological activity and modification. Here, we summarize the current knowledge on the extraction, purification, modification, and biological activities of polysaccharides from TCMs.

## 2. Extraction of TCM Polysaccharides

### 2.1. Extraction Pre-Processing

TCM polysaccharides are classified into two types according to their locations in the plant: intracellular polysaccharides (IPSs) and extracellular polysaccharides (exopolysaccharides, EPSs) [20]. To facilitate the release of IPSs, the first step of extraction is mechanical crushing of the TCM material. Next is degreasing, as many plant seeds and animal products are rich in lipids, which largely surround the cell wall. Usually this is accomplished by Soxhlet extraction using an organic solvent because of the hydrophilicity of the polysaccharides. Subsequently, the polysaccharides are extracted by means of various methods.

### 2.2. Extraction Method

The basic extraction principle is to destroy and degrade the cell wall under mild conditions so that the intrinsic properties of polysaccharides remain unchanged [21,22]. Traditional water extraction (TWE) is a common and popular approach for polysaccharide extraction, especially hot water extraction (HWE), which is the most traditional and classic one. Recently, various novel techniques have been developed, including enzyme-assisted extraction (EAE) [23], microwave-assisted extraction (MAE) [24], ultrasonic-assisted extraction (UAE) [25], and supercritical fluid extraction (SFE) [26]. An appropriate extraction method is not only a means to increase the extraction yield, but also contribute to the high biological activity of the resulting polysaccharide extract [27]. Every extraction method has its strengths and weaknesses, so a comprehensive understanding of these effects on the physical and chemical properties of the polysaccharide is necessary.

#### 2.2.1. Traditional Water Extraction

In general, TCM polysaccharides are polar and hydrophilic macromolecules. Polysaccharides can be extracted with a strong polar solvent, and water is an ideal solvent which is popularly used in practice. High temperature can accelerate the dissolution of polysaccharide from the cell wall and make it easier to dissolve in water [28]. Thus, hot water extraction (HWE) has long been the traditional and classic method for polysaccharide extraction [22]. Many studies have demonstrated that the extraction yield of HWE is largely affected by extraction time, temperature, ratio of water to raw material, and the number of extraction steps [29,30,31,32]. Especially, HWE of long duration and at high temperature may result in the degradation of the polysaccharides and decrease the biological activity [22,27]. For example, the yield of polysaccharides from the roots of *Codonopsis pilosula* increases up to its maximum amount at 94°C, but beyond this level, the yield decreases with increasing extraction temperature [33]. Usually, the common temperature range for HWE is between 70 °C and 90 °C, and the extraction time is between 2 h and 6 h, which are suitable for most polysaccharides [3]. In a word, HWE is the most common and convenient method to extract polysaccharides, and some of its major drawbacks are the high temperatures, long times, and low efficiency.

To improve the efficiency and purity, dilute alkaline and acidic solution methods based on HWE have come into use. Some acidic polysaccharides are not easily dissolved in hot water, and therefore an alkaline solution is applied to increase their solubility [3]. An acidic polysaccharide could be isolated from *Morinda officinalis* by alkaline solvent extraction [34]. However, the glycosidic bonds of polysaccharides easily break under acidic conditions, so acidic solutions are seldom used. The extraction pH, time, and temperature should be limited to a certain range, or the structure of the polysaccharide could be destroyed.

#### 2.2.2. Enzyme-Assisted Extraction

Owing to the relatively mild reaction conditions, EAE possesses the advantages of environmental friendliness, high efficiency, ease of operation, low investment cost and energy requirements [23,35]. EAE involves the use of enzymes, such as proteases, cellulases, amylases, glucanases, or endoproteases [35], which effectively and mildly catalyze the degradation of the cell wall and facilitate the release of IPSs [36,37]. However, the enzyme is characterized by specificity and selectivity while, at the same time, the biological activity is influenced by several factors, such as enzyme concentration, temperature, time, and pH, which cooperatively affect the extraction [38,39]. Thus, only one kind of enzyme usually cannot satisfy the extraction objectives. Composite enzymes or a cocktail of enzymes which has a broad spectrum of activity is one of the best methods to disrupt the cell wall [40]. Moreover, it can lead to a higher level of hydrolysis because of synergistic effects. It has been reported that an efficient complex enzyme-assisted extraction technology has been developed and optimized to extract polysaccharides from alfalfa [41]. As is known to all, every enzyme has optimum reaction conditions where the activity is the highest, while the optimal condition of composite enzymes could be different from that of any single enzyme [42]. Thus, the synergistic effects, types of substrate and the presence of any enzyme inhibitors also should be considered in EAE. EAE is seldom used alone, and is usually combined with other extraction methods to increase the yield of polysaccharide.

#### 2.2.3. Physical Technique-Assisted Extraction

Physical techniques have demonstrated their extraction ability. In comparison to traditional extraction methods, the yield of polysaccharides increases with lower cost and shorter time [43]. There are three important methods commonly used in research and practice.

MAE has drawn significant attention in the analysis and extraction of active compounds from TCM materials [44]. Some studies have suggested a mechanism whereby microwave power rapidly ruptures the cell structure and releases the intracellular products into the solvent [45,46,47]. On the other hand, microwaves can also penetrate into products and interact with polar components to generate heat [44]. The resulting kinetic energy and high temperature will accelerate the mass transfer in a short time [44,48]. Due to its special mechanism, MAE has noticeable advantages, such as shorter extraction times, higher extraction yields, lower cost, and less solvent consumption compared to TWE [49,50,51,52,53], and factors including microwave power, extraction time, ratio of solid to solvent, and microwave application duration may influence the efficiency of MAE [54,55]. Previous studies have reported the possibility of polysaccharide decomposition during MAE [56,57]. Furthermore, there are some drawbacks associated with MAE, such as the requirement of additional clean up steps and the restriction to polar solvent application [58]. However, it is apparent that the technique of MAE has been continuously improved to enhance its performance.

The use of UAE as an alternative and promising method has been on the rise. As a tool for extraction intensification, the mechanism of UAE is not yet well understood [59,60]. Many studies have reported that the possible benefits of ultrasound may be due to an enhancement of the mass transfer between the plant and solvent [61,62,63], intensification of shear forces arising from acoustic cavitation [20,64], and improved penetration and capillary effects [65]. All of these lead to an increase of polysaccharides’ extractability by destruction of the cell wall [54,62]. On the other hand, ultrasonic treatment could affect the structure and molecular weight (MW) of polysaccharides to some extent, which would inevitably cause a change in the biological activity [66,67,68,69]. Several parameters are involved in UAE, such as the type of ultrasonic device, the frequency and power of the ultrasound, and the sonication time [43].

SFE is a new method developed in recent years to extract polysaccharides. Supercritical fluids are a special kind of solvent, and has the characteristics of both liquid and gas which can effuse through solids like a gas, and dissolve materials like a liquid. SFE increases the solubility of polysaccharides and enables their better extraction [70]. CO_2_ is often used as the solvent for SFE because its critical conditions is easy to achieve. Pachyman in *Poriacocos (Schw.) wolf* has been extracted by SFE under optimal conditions involving an extraction temperature of 35 °C and CO_2_ pressure 20 MPa [71]. The main advantages of SFE over TWE are the improvement of extraction efficiency and shorter production cycle, as well as the elimination of polluting solvents and expensive post-processing [26]. However, the problems of SFE are complex equipment requirements, high operational cost, and limited applicability.

## 3. Purification of Polysaccharides

### 3.1. Removal of Impurities

Generally the extract is a mixture which contains proteins, pigments, small molecules, and other impurities. Therefore, a subsequent processing step should be performed to remove these impurities. The common deproteinization methods are the Sevag method [72], the trichlorotrifluoroethane method [3], and the trichloroacetic acid method [73]. In these methods, the reagents used for denaturation and precipitation of protein hardly work for polysaccharides, so the protein should be removed. For example, after addition of the Sevag reagent (chloroform: *n*-butanol = 4:1) to polysaccharide aqueous solution, the mixture is violently shaken and centrifuged. Usually this process is repeated five times or more to get rid of the protein. In order to obtain a better removal rate, several methods can be combined together to reduce time and solvent use at the same time. Pigments always affect the purification and property analysis of polysaccharides. They can be absorbed with activated carbon, resins, or destroyed by hydrogen peroxide treatment [73,74]. However, activated carbon can absorb the polysaccharide causing a yield loss, and hydrogen peroxide treatment can also cause partial hydrolysis of the polysaccharide [75,76]. In general, these two methods are seldom used for decolorization. A polysaccharide is macromolecule, so small molecules, like oligosaccharides and inorganic salt, can be easily removed using dialysis against water. After these treatments, the resulting extract still needs to be purified further to obtain pure polysaccharide in composition and properties.

### 3.2. Purification Method

Most purification methods are based on solubility differences, and the fractionated precipitation technique is considered as a unique and convenient approach to selectively yield polysaccharides with large differences in solubility in lower alcohols or ketones [77]. In this method, the lower alcohol or ketone is gradually added to the extract. Then the precipitated polysaccharides at each concentration of the lower alcohol or ketone can be collected by centrifugation. With the increasing concentration, the MW of obtained polysaccharides gradually reduces. It has been reported that three main polysaccharide fractions from *Asparagus officinalis*, AOP-4, AOP-6, and AOP-8 are obtained by fractional precipitation using gradient concentrations of ethanol (40%, 60%, and 80%) [78]. The salting-out method is also able to separate different polysaccharides out in the form of a precipitate [3]. Furthermore, long-chain quaternary ammonium salts and various metal ions can form coordination compounds with polysaccharides to achieve separation and purification.

On the other hand, the chromatography technique is one of the most effective separation and analysis methods. This technique can separate out individual polysaccharides from the extract with high sensitivity, selectivity, and separation resolution. The most used methods to purify polysaccharides are gel permeation chromatography and ion exchange chromatography. Gel permeation chromatography (size exclusion chromatography) is based on the simple principle of the separation of molecules due to size [79]. Briefly, the large molecules simply pass by the pores of particular gels because those molecules are too large to enter the pores, while the small molecules are trapped in the pores. Therefore, high MW polysaccharide is eluted out first, and then low MW polysaccharide is eluted. Various gels have been used, like Sephacryl S-400 HR, Sephadex G-200, and Sepharose 6B [80]. Most TCM polysaccharides are weak acids, so they are charged by ionic complex formation in aqueous media [81]. Ion exchange chromatography is able to separate acidic and neutral polysaccharides based on their net charge differences. The ionizable molecules can bind to oppositely charged moieties by forming covalent bonds to the insoluble stationary phase, and then these molecules can be gradually eluted under different elution conditions. In this way, the polysaccharides can be separated and quantified. For example, the polysaccharides extracted from the dried fruiting bodies of *Pithecellobium dulce* have been isolated by ion exchange chromatography and afford three water-soluble polysaccharides PDP-1, PDP-2, and PDP-3 [82].

Furthermore, other techniques like ultracentrifugation, ultrafiltration [83], and preparative zone electrophoresis can also be applied to the purification of polysaccharides. To achieve high purity of polysaccharides, many researchers advise using two or more methods simultaneously.

## 4. Analysis of Polysaccharides

It is generally accepted that the biological activity of polysaccharides often depends on the molecular structure and other physicochemical properties, including water solubility, MW, monosaccharide composition, glycosidic bonds of the main chain, etc. [84], so the composition of polysaccharides is critical to their biological activity. Since many kinds of proteins, lipids, biological bases, and other ingredients usually exist together with polysaccharides in TCM materials, the analysis is very difficult. Therefore, the extraction and purification can greatly influence the analysis. At present, there are various methods for the analysis. The total content of polysaccharide is often determined by colorimeter with different chromogenic systems, such as phenol-sulfuric acid [85,86], anthracenone-sulfuric acid [87], and carbazole-sulfuric acid [88]. As the strongest separation technique, chromatographic methods have been extensively applied to the compositional and structural analysis of polysaccharides when combined with other structural analytical techniques, including IR, NMR, MS, and so on [81]. According to the previous studies [80,81,89,90], the different chromatography techniques are shown in Table 1. TCM polysaccharides are complicated and the pretreatments must be done before analysis. In general, there are a number of ways to pretreat polysaccharides, including Smith degradation, periodate oxidation, methylation analysis, etc., and then the various analysis techniques mentioned above can be applied to the compositional and structural determination. Moreover, the electromigration method has very high separation efficiency and is also extensively used for analysis of polysaccharides. With the emergence and development of modern analysis techniques, more and more methods will be created and introduced to the analysis of polysaccharides.

## 5. Modification of Polysaccharides

Chemical modification is an important approach to tailor polysaccharide structure and properties [91]. In the last decades, much attention has been focused on the modification of polysaccharides which can heighten or change the biological activities. Among the various modification methods, sulfation, phosphorylation, and carboxymethylation are the most common and important.

### 5.1. Sulfation

It is well known that sulfation of polysaccharides is an effective method to modify their biological activity and function. In general, the hydroxyl groups of TCM polysaccharides are substituted by sulfate groups [92], and it has been reported that the hydroxyl groups of the monosaccharide residues at C-6 are more active owing to the steric hindrance effect and the absence of a hydrogen bond, therefore substitution at C-6 easily occurs [93,94]. There are several methods for polysaccharide sulfation, using different reagents such as sulfuric acid, sulfur trioxide-pyridine [95], chlorosulfonic acid-pyridine (CSA-Pyr), and so on [96]. The first two are less used because of low degree of esterification and recovery. CSA-Pyr, by contrast, is the most common and widely used one since it is characterized by high yield and convenient isolation of the product [97], and it has been applied in the sulfation of polysaccharides from *Cyclocarya paliurus* [85], *Cyclina sinensis* [98], *Borojoa sorbiliscuter* [99], etc. The sulfation can affect the MW, charge, solubility, and conformation of the polysaccharide [100]. As a result, the biological activity will be heightened or changed. There are many reports demonstrating the influence of sulfation on the biological activities, including antioxidant, immunostimulant, antitumor, antiviral, anticoagulant, hypoglycemic, and cytotoxic properties [101,102,103,104,105,106,107,108]. For example, sulfated *Angelica* polysaccharides-1 could not only inhibit virus replication, but also improve the immune function. Additionally, the activity of sulfated polysaccharide also depends on structural modification, such as the degree of substitution (DS), the sulfation position, the type of polysaccharide, and the structure of the main chains and branches [99,109,110,111]. In particular, the DS which represents the average number of sulfate groups on each monosaccharide residue [92,112] is significantly important to the activity. Previous studies have shown that there is an optimum DS of sulfation to achieve a maximal biological response [101]. The antiviral activity of sulfated polysaccharides reaches the best value when the DS was within the range of 1.5–2.0 [113].

After sulfation, it is also needed to confirm whether the modification was successful. For sulfate estimation, the common method is the turbidometric barium chloride method [114,115], and the sulfur content is estimated using the benzidine method [116] and elemental analysis [92]. The DS of sulfation can subsequently be calculated using the formula: DS= 1.62 × S/(32 −1.02 × S), where S is the sulfur content of sulfated polysaccharide [117]. Infrared spectroscopy (IR) and nuclear magnetic resonance (NMR) are often used to analyze the structure changes caused by modification. Generally, the signals of the polysaccharide backbone chain still exist after modification, which indicates that the main structure of the derivative is preserved, and some new or changed signals appear, suggesting the modification changes. Compared with *Artemisia sphaerocephala* polysaccharide, two characteristic absorption bands appear in the IR-spectrum of sulfated *Artemisia sphaerocephala* polysaccharide near 1250 and 810 cm^−1^ indicating the sulfation reaction has occurred. As for the NMR results, the new signal at 67.0 ppm of sulfated *Artemisia sphaerocephala* polysaccharide could be assigned to the O-6 substituted carbons, suggesting sulfation at O-6 [118].

### 5.2. Phosphorylation

The phosphorylation of polysaccharides is essentially an esterification process [112], and it is also a kind of covalent modification, during which the hydroxyl groups of the branched chain re substituted by a phosphate group [119]. Until now, many methods have been reported to synthetize phosphorylated derivatives, such as the polyphosphoric acid/tributylamine/dimethyl formamide method [120], H_3_PO_4_/fatty acid esters method [121], and the H_3_PO_4_/palmitic acid/tertiary amines method [122]. As for TCM polysaccharides, the common reagents used are phosphate, phosphorus oxychloride, and phosphoric anhydride, and the phosphorylation of different TCM polysaccharides is listed in Table 2. After modification, the physicochemical and biological properties of polysaccharides are changed [123]. For example, phosphorylation may contribute to the improvement of water solubility. A water-soluble polysaccharide is generated through phosphorylation of *Dictyophora indusiata* polysaccharide which is, conversely, water-insoluble [123]. It is noted that phosphorylation can enhance the anti-oxidative [124], anti-tumor [93], and antiviral [125] activities. The studies on other activities of phosphorylated TCM polysaccharides, like anti-inflammatory, antibacterial, and anticoagulant abilities [126], are relatively scarce. However, the phosphorylation does not always improve the activity of the polysaccharide. Phosphorylated porphyran shows weaker inhibitory effect on the superoxide radical than porphyran [127].

Like sulfation, the extent of phosphorylation also needs to be tested. Firstly, the total phosphate content in the derivative can be determined by the ascorbic acid method [128], the phosphate group content can be determined by the molybdenum blue method [129,130], and the phosphorus contents can also be determined by ICP-AES [131]. Moreover, the structures of phosphorylated derivatives can also be analyzed by IR and NMR spectroscopy. The bands in the IR-spectrum polysaccharide from *Porphyra haitanensis* at 1268 cm^−1^ and 988 cm^−1^ indicate P=O stretching vibrations and P–O vibrations, respectively [127]. To confirm the introduction of the phosphate group, ^31^P-NMR and ^13^C-NMR are widely applied to the study of polysaccharide structure [123].

### 5.3. Carboxymethylation

Carboxymethylation is another versatile modification method. Due to the substitution of one hydrogen atom from the OH group by a carboxymethyl group [132,133,134], it is able to change the biological activity of TCM polysaccharides. The common method for carboxymethylation is to dissolve the polysaccharide in an alkaline solution, and add a certain amount of chloroacetic acid to react under proper conditions, then adjust to neutral pH using hydrochloric acid or acetic acid. After purification, the carboxymethylated derivative is obtained. The introduction of the carboxymethylated group brings new activity or enhances the intrinsic activity of the polysaccharide [135,136,137]. For example, *Poria sclerotium* polysaccharide without anti-gastric adenocarcinoma activity shows strong anti-gastric adenocarcinoma activity after carboxymethylation [138], and the carboxymethylated derivative exhibits stronger antioxidant activity compared to the polysaccharide from *Auricularia auricular* [139]. Additionally, there are other studies on the improvement of antitumor, immune enhancement, etc. [140].

To confirm the success of carboxymethylation, DS can be determined using the colorimetric method [139,141], conductometric titration [142], the acid-washed method, and neutralization titration. In addition, DS is able to be affected by chloroacetic acid concentration, reaction time, and temperature [143]. As for the structure, IR and NMR spectroscopy can also be applied. Two new intense absorption bands of carboxymethylated *Ganoderma applanatum* polysaccharide in the IR-spectrum at 1623 cm^−1^ and 1333 cm^−1^ correspond to the asymmetrical and symmetrical stretching vibrations of the COO^−^ group, and a new resonance signal appearing at 176.90 ppm in the NMR spectrum is assigned to the carboxylate group, which provides strong evidence of carboxymethylation [144].

### 5.4. Other Modifications

In addition to the above three modifications, other methods, including acetylation, selenylation, alkylation, and benzoylation [145] are also used to modify TCM polysaccharides. These methods not only improve the water solubility but also heighten or change the biological activities. For example, selenylation can improve the immune-enhancing activity of natural polysaccharides [146]. However, the reagents used, like Na_2_SeO_3_ and SeOCl_2_, are toxic and are threats to human health. Since these modifications have certain limitations or are little studied, the details will not be covered in this review.

## 6. Biological Activities of TCM Polysaccharides

### 6.1. Immunity

In TCM theory, disease prevention treatment is the most important objective and it strengthens our bodies’ disease resistance by kinesiotherapy and TCM, which relate to immunity. Almost all of TCM polysaccharides possess immunoregulatory effects [3,147,148,149,150], and various biological activities of TCM polysaccharides are seemingly related to their immunoregulation effects. This may be because polysaccharides are one of the main identification objects of the immune system.

#### 6.1.1. Immune Cell

T-lymphocytes are an important type of immune cell which play a pivotal role in the immune system through cellular immunity. A lot of TCM polysaccharides are able to increase the number and activity of T-lymphocytes. *Grifola frondosa* polysaccharide D [151] is able to increase the activity and the amount of CD8^+^ T cells. In the meantime, it induces the TH1 immune pathway and increases IL-2 secretion. Bush sophora root polysaccharide accelerates the proliferation of T lymphocytes and its sulfate enhances such ability [4].

TCM polysaccharides are also able to improve the amount and activity of another important immune cell type, B lymphocytes. *Astragalus* polysaccharide [152], epimedium polysaccharide [153], and Bush sophora root polysaccharide [4] all accelerate the proliferation of B-lymphocytes. The binding of *Acanthopanax senticosus* polysaccharide and toll-like receptor stimulates mitogen-activated protein kinases and then promotes the growth and differentiation of B-lymphocytes [154].

Natural killer cells and macrophagocytes are also vital immune system cells. Similarly, TCM polysaccharides, such as *Lentinus edodes* polysaccharide [155] and *Tinospora cordifolia* polysaccharide [156], are able to stimulate the activities of natural killer cells and macrophagocytes.

#### 6.1.2. Cytokines

Cytokines are secreted by immune and non-immune cells and play significant roles in immunoregulation. TCM polysaccharides regulate the immunity by their influence on the secretion of cytokines. *Ganoderma lucidum* polysaccharide is able to promote the secretion of IL-1, IL-6, IFN-γ, TNF-α, GM-CSF, GVCSF, M-CSF, and IL-12 [157]. *Astragalus* polysaccharide modulates the expression of TNF-α, IL-1β, and NFATc4 in a rat model of experimental colitis [10]. *Angelica sinensis* polysaccharide stimulates the production of IFN-γ, IL-2, IL-6, and TNF-α [158]. *Cordyceps militaris* is a rare kind of Chinese traditional medicine; its polysaccharide can up-regulate the expression of TNF-α in mouse peritoneal macrophages and RAW264.7 macrophages [159].

#### 6.1.3. Antibody

Antibodies, including IgM, IgG, IgA, IgE, and IgD, are important kinds of immunoglobulins. Often, the antiviral activity of a TCM polysaccharide is due to its promoting effect on antibodies. Bush sophora root polysaccharide reduces the amount of duck hepatitis A virus by stimulating the antibodies [4]. Both *Lycium barbarum* polysaccharide [160] and *Rehmannia glutinosa* polysaccharide [161] exhibit anti-porcine circovirus type 2 effects by promoting the specific IgG.

#### 6.1.4. Complement System

Activation of the complement system enhances immunity. Some TCM polysaccharides exhibit complement activation. Polysaccharides from *Vernonia kotschyana* Sch. Bip. ex Walp exhibit strong complement activation abilities [162]. Though the complement system is one of the important immune defense systems, excessive activation can result in too strong an inflammatory response and diseases, such as rheumatoid arthritis. Polysaccharides, like those from the root of *Bupleurum chinense* [163] and the stem of *Ephedra sinica* Stapf [164], exhibit anti-complement effects.

### 6.2. Antiviral Activity

Antiviral activities of polysaccharides are well known and such activities of TCM polysaccharides are affirmed by many studies (Table 3). As far back as 1958, Gerber et al. [165] reported the anti-influenza B and mumps virus activities of polysaccharides extracted from *Gelidium cartilagenium*, which is called “qilincai” in TCM. *Astragalus* polysaccharide shows antiviral activities against infectious bursal disease virus [166], porcine circovirus virus type 2 [12], duck hepatitis A virus [167], human hepatitis B virus, porcine reproductive and respiratory syndrome virus, and classical swine fever virus [22].

It is generally recognized that the antiviral activities of TCM polysaccharides are related to the activation of the immune system, such as activating macrophagocytes to promote their phagocytic ability and inducing the secretion of IL-2, IFN-γ, and antibodies [4,155,157,160,167]. Antiviral activities of TCM polysaccharides are also related to sulfate radicals. It is reported that sulfate modification of *Astragalus* polysaccharide [167] and Bush sophora root polysaccharide [17] increases the inhibitory rate of duck hepatitis A virus, and *Chuanminshen violaceum* polysaccharide exhibits no anti-duck enteritis virus activity. After sulfate modification, sulfated *Chuanminshen violaceum* polysaccharide possesses significant antiviral activity against duck enteritis virus, ranging from 77.12 μg/mL to 104.81 μg/mL [169]. Additionally, TCM polysaccharides are able to block different stages of viral life. Sulfated *Chuanminshen violaceum* polysaccharide inhibits the adsorption of duck enteritis virus on duck embryo fibroblasts [169]. Bush sophora root polysaccharide inhibits the replication of duck hepatitis A virus, and its sulfate inhibits replication and release [17].

### 6.3. Anti-Inflammatory Activity

Inflammation is a common and important pathological process. It is body’s defense response to stimulus. The anti-inflammatory activity of TCM polysaccharides is mainly due to the inhibition of the expression of the chemotactic factor and adherence factor, as well as the activities of key enzymes in the inflammation process. CCl_4_ causes an inflammatory response by increasing TNF-α and IL-1β. Treatment with *Ganoderma lucidum* polysaccharides in pre-treatment and post-treatment modes attenuates the increases of TNF-α and IL-1β [175]. *Lycium barbarum* polysaccharide alleviates the inflammatory reaction in the kidneys of diabetic rabbits through reducing the expression of MCP-1mRNA and ICAM-1mRNA by restraining the expression of NF-κB and AngII [176]. *Astragalus* polysaccharide reduces cell accumulation and swelling, the arthritic index of joints, and serum concentrations of TNF-α and IL-1βin adjuvant-induced arthritic rats [22].

### 6.4. Anti-Oxidative Activity

Under normal circumstances, free radicals regulate cell growth as well as inhibit viruses and bacteria. However, many free radicals, including superoxide anion, hydrogen peroxide, and NO, also cause various types of diseases, such as cancer, arteriosclerosis, and aging [177]. Biological antioxidants can prevent and cure these diseases by breaking peroxide chain reactions. A lot of studies show that TCM polysaccharides possess anti-oxidative abilities [12,178,179,180,181,182,183].

TCM polysaccharides are able to scavenge free ROS [12,182,183]. Each monosaccharide unit possesses active hydroxyl groups which can combine with hydroxyl radicals or superoxide anion free radicals to form water. In addition to this, it is also related to some other mechanisms. *Astragalus* polysaccharide [12] and *Rhizoma Dioscoreae* Nipponicae polysaccharide [184] inhibit ROS generation by blocking the NF-κB pathway. Inhibition of ROS induced by angiotensin II of *Bletilla striata* polysaccharide is involved in the NOX4 pathway [182]. *Cordyceps sinensis* polysaccharide inhibits PDGF-BB-induced inflammation and ROS production via the ERK/Akt pathways [183].

The anti-oxidative activity of TCM polysaccharides is also due to their promoting effect on antioxidant enzyme activity. The main antioxidant enzymes in bodies are SOD, CAT, and GSH-Px. Bush sophora root polysaccharide is able to increase the activities of SOD, CAT, and GSH-Px [178]. *Lycium barbarum* polysaccharides increase the activities of GSH-Px and SOD in chickens [181]. The anti-oxidative activity of *Astragalus* polysaccharide has been widely studied [12,167], and the results show that the anti-oxidative activity of *Astragalus* polysaccharide is mainly attributable to the enhancement of antioxidant enzyme activity [12,167,170].

### 6.5. Anti-TumorActivity

Cancer is a severe human disease. Chemotherapy and radiotherapy, as is well-known, are the primary treatment methods, however, normal cells are injured when tumor cells are killed by chemotherapy or radiotherapy. Therefore, research on anti-tumor drugs with low toxicity and high efficiency is vitally important. A lot of studies in recent years show that many TCM polysaccharides possess anti-tumor activities with low toxicity (Table 4).

TCM polysaccharides play anti-tumor roles through two aspects. One is by inducing cell apoptosis or inhibiting the expression of cellular oncogenes to kill tumors directly. *Lycium barbarum* polysaccharide [185] and *Angelica sinensis* polysaccharide [186] kill tumor cells by inducing apoptosis. *Astragalus* polysaccharide [187,188] directly reduces the mass of tumors in vivo. *Radix Hedysari* polysaccharide and *Achyranthes bidentata* polysaccharide are able to arrest the cell cycle and thus play anti-tumor roles. Another anti-tumor mechanism is by enhancing the immunity to kill tumor cells indirectly. In this mechanism, TCM polysaccharides do not act on tumor cells, but rather they play the role by activating the immune system. For example, *Sophora flavescens* polysaccharide [189], *Sanguisorba officinalis* polysaccharide [190], *Curcuma kwangsiensis* polysaccharide [191], *Schisandra chinensis* polysaccharide [192], and *Ganoderma lucidum* polysaccharide [193] all enhance the immunity system so that the growth of tumors is inhibited. As TCM polysaccharides possess characteristics of low toxicity and side effects, as well as high anti-tumor efficiency, they are expected to be a new kind of anti-tumor drug.

### 6.6. OtherActivities

TCM polysaccharides also possess hypoglycemic, neuroprotective, hair growth promoting, anti-vomiting, anti-glaucoma, hepatoprotective, hematopoietic, growth-promoting, antiatherosclerotic activities, and so on [3,170].

## 7. Summary

Conspicuously, TCM polysaccharides possess various abilities to enhance resistance or treat diseases, and the potential applications of TCM polysaccharides in the clinic are excellent. However, the extraction, purification, and modification often affect the activities of homogeneous TCM polysaccharides. Thus, studies must choose appropriate methods of extraction, purification, and modification, and the particular action mechanisms of specific TCM polysaccharides still need to be studied further.

## Figures and Tables

**Table 1:** The comparison of different chromatography techniques.

Technique	Application	Advantage	Disadvantage
Gas chromatography, GC	Determination of dissociated monosaccharides, and constituent monosaccharides of oligosaccharides and polysaccharides	Simple instrumentation, high selectivity, high resolution, high accuracy	Limited to volatile compounds, thermal instability
High-performance liquid chromatography, HPLC	Purification, qualitative and quantitative determination of dissociated monosaccharides, oligosaccharides, and constituent monosaccharides of polysaccharides, MW determination	High separation efficiency, wide availability, high sensitivity and reproducibility, good resolution and linearity, high accuracy and precision	Extended analytical time
Ultra-performance liquid chromatography, UPLC	Similar to HPLC as an advanced technique	Reduced analytical time, reduced solvent consumption	Reduced solvent consumption, high equipment requirement
Gel column chromatography, GCC	Purification and MW determination of polysaccharides	Quickness, high selectivity, good repeatability	Low mass resolution
Thin layer chromatography, TLC	Purity measurement, determination of constituent monosaccharides of oligosaccharides and polysaccharides	Simple instrumentation, versatility, quickness, flexibility and low cost	Low efficiency in separation, low accuracy and sensitivity, unsatisfactory repeatability

**Table 2:** The phosphorylation of TCM polysaccharides.

Polysaccharide Source	Phosphyorylation Reagent	Analysis Result	Activity Comparison	Ref.
*Cucurbita pepo*	Phosphorus oxychloride/pyridine	DS: 0.33–0.52	Higher antioxidant activity	[19]
*Enteromorpha linza*	Formamide/tributylamine/polyphosphoric acid	Phosphate (%): 4.05	Higher antioxidant activity	[122]
*Porphyra*	Formamide/tributylamine/polyphosphoric acid	Phosphate (%): 4.33	Parallel scavenging activity	[127]
*Dictyophora indusiata*	Orthophosphoric acid/urea	DS: 0.206	Higher water solubility, antioxidant, and antitumor activities	[123]
*Cyamopsis tetragonoloba*	Triphenylmethyl chloride/phosphorus oxychloride/pyridine	DS: 0.34	Higher antioxidant activity	[110]
*Polygonatum cyrtonema* Hua	Phosphorus oxychloride/triethyl phosphate/ pyridine	DS: 0.65	Higher antiherpetic activity	[125]
*Poria cocos*	Urea/dimethyl sulfoxide/orthophosphoric acid	DS: 0.056–0.153	New antitumor activity	[131]
*Portulacao leracea* L.	Dimethyl formamide/3-phosphono-propionic acid/catalyst	DS: 0.09–0.33	Higher antioxidant activity	[112]

**Table 3:** Antiviral activities of TCM polysaccharides.

Virus Species	Virus	Polysaccharide Source	Ref.
ssDNA viruses	Porcine circovirus virus type 2	*Astragalus*	[12]
		*Lycium barbarum*	[160]
		*Sophora subprosrate*	[168]
		*Rehmannia glutinosa*	[161]
dsDNA viruses	Duck enteritis virus	*Chuanminshen violaceum*	[169]
DNA reverse transcribing viruses	Human hepatitis B virus	*Astragalus*	[170]
		*Chickweed*	[171]
(+)ssRNA viruses	Duck hepatitis A virus	Bush sophora root	[17]
		*Astragalus*	[167]
	Porcine reproductive and respiratory syndrome virus	*Astragalus*	[170]
		*Achyranthes bidentata*	[172]
	Swine fever virus	*Astragalus*	[170]
(−)ssRNA viruses	Influenza B virus	*Gelidium cartilagenium*	[165]
	Newcastle disease virus	*Chuanmingshen violaceum*	[173]
		*Lycium barbarum*	[106]
		*Ophiopogon japonicus*	[174]
	Mumps virus	*Gelidium cartilagenium*	[165]
dsRNA viruses	Infectious bursal disease virus	*Astragalus*	[166]
		Epimedium	[106]

**Table 4:** Anti-tumor activities of TCM polysaccharides.

Aspects	Polysaccharide Source	Tumor cell	Anti-Tumor Activity Mechanism	Ref.
Directly	*Astragalus*	H22, HepG2, MGC-803	Inhibit growth of tumor	[187,188]
	*Lycium barbarum*	MCF-7, BIU87	Induce apoptosis	[185]
	*Radix Hedysari*	A549, BGC-823	Arrest the cells at G1 phase	[194]
	Centipede	S180, H22	Inhibit growth of tumor	[195]
	*Angelica sinensis*	Hela	Induce apoptosis	[186]
	*Achyranthes bidentata*	LLC	cell cycle arrest	[196]
	*Cordyceps sinensis*	H157	Inhibit growth of tumor	[197]
Indirectly	*Sophora flavescens*	H22	Enhance immunity	[189]
	*Sanguisorba officinalis*	S180	Enhance immunity	[190]
	*Curcuma kwangsiensis*	CNE-2	Enhance immunity	[191]
	*Schisandra chinensis*	L5178Y	Enhance immunity	[192]
	*Ganoderma lucidum*	MDA-MB-231	Enhance cytophagy effect of macrophagocyte	[193]
